# Genome-wide analysis identifies common CNVs associated with primary open angle glaucoma

**DOI:** 10.1186/1755-8166-7-S1-P131

**Published:** 2014-01-21

**Authors:** Lalit Kaurani, Mansi Vishal, Dhirender Kumar, Bharati Mehani, Charu Sharma, Anchal Sharma, Debasis Dash, Jharna Ray, Abhijit Sen, Kunal Ray, Arijit Mukhopadhyay

**Affiliations:** 1 Genomics & Molecular Medicine, CSIR-Institute of Genomics & Integrative Biology, Delhi, India; 2 Molecular & Human Genetics Division, CSIR-Indian Institute of Chemical Biology, Kolkata, India; 3 G. N. Ramachandran Knowledge Centre for Genome Informatics, CSIR-Institute of Genomics & Integrative Biology, Delhi, India; 4 Mathematics Department, School of Natural Sciences, Shiv Nadar University, Uttar Pradesh, India; 5 Academy of Scientific and Innovative Research (AcSIR), Delhi, India; 6 S. N. Pradhan Centre for Neurosciences, University of Calcutta, Kolkata, India; 7 Drishti Pradip Eye Clinic, Kolkata, India

## Background

Copy number variation (CNV) is one of the major factors contributing to genomic diversity and diseases. Glaucoma is a major neurodegenerative disease causing irreversible vision loss across the globe. We wanted to analyze the impact of common CNVs in a genome-wide scale in patients of primary open angle glaucoma (POAG) collected from the West Bengal, India.

## Method

Genome-wide data was generated on 364 POAG cases and 365 controls on Illumina 660W-Quad arrays and CNVs were called using PennCNV. Copy number variant regions (CNVRs) were analyzed for association. A publicly available dataset of POAG cohort of 866 cases and 495 controls from Caucasian origin (GLAUGEN study) was used as a validation cohort. Representative CNVs were validated using real-time PCR.

## Results

We analyzed genome-wide CNV from 1928 samples. After association analysis we found 308 significantly associated (p<0.05) CNVRs in the Indian data. These POAG associated CNVRs were enriched in nervous system development. 113 CNVRs (37%) were significantly associated with the Caucasian data set. These contain 5 genes previously reported in eye diseases, namely, *IDUA*, *FOXE3*, *NDUF7*, *PRPF6* and *WNT3*. We also found 6 associated CNVRs in previously known glaucoma loci.

## Conclusion

We have shown that common CNVRs are significantly associated in both datasets irrespective of the population background. We have also identified candidate genes/regions which are uniquely present in POAG cases and absent in controls. Our data might provide new insights into role of CNV in pathogenesis of POAG.

